# Antioxidative effect of loquat (*Eriobotrya japonica* Lindl.) fruit skin extract in soybean oil

**DOI:** 10.1002/fsn3.193

**Published:** 2014-12-18

**Authors:** Mojtaba Delfanian, Reza Esmaeilzadeh Kenari, Mohammad Ali Sahari

**Affiliations:** 1 Department of Food Science and Technology, Sari Agriculture and Natural Resources UniversitySari, Mazandaran, Iran; 2 Department of Food Science and Technology, Faculty of Agriculture, Tarbiat Modares UniversityTehran, Iran

**Keywords:** *Eriobotrya japonica*, solvent extraction, soybean oil, supercritical fluid, ultrasound

## Abstract

The aim of this study was to compare the effects of solvent and ultrasound-assisted extraction methods with supercritical fluid extraction on antioxidant activity of loquat (*Eriobotrya japonica* Lindl.) fruit skin extract in stability of soybean oil at 25°C. Oxidative stability alterations of soybean oils containing 400 (SEA) and 1000 ppm (SEB) of ethanol extract, 400 (SSA) and 1000 ppm (SSB) of supercritical CO_2_ extract, 400 (SUA) and 1000 ppm (SUB) of ultrasound-assisted extract, and 100 ppm of tertiary butylhydroquinone (TBHQ) were monitored by measuring the peroxide value, thiobarbituric acid value, free fatty acids, conjugated dienes and trienes values. Oxidative changes in SEA were lower than that of oils treated with other extracts, but the best protection was observed in soybean oil consisting TBHQ. The solvent extraction method produces the maximum amount of phenolic and tocopherol compounds from loquat fruit skin. Therefore, solvent extraction method had a better effect on antioxidant activity of the loquat fruit skin extract.

## Introduction

Oils and fats undergo oxidative changes during storage conditions which cause reduction in the nutritional quality and shelf life of them (Mohdaly et al. 2010). The compounds produced during the oxidation of oils and fats such as hydroperoxide, hydroxyl radical, and single oxygen capacity can do more damage to biological molecules (Pincemail et al. 2002). This causes cellular damage and the development of physiological abnormalities in human, such as premature age, heart disease, and neurological (Suja et al. 2005).

The synthetic antioxidants, such as butylated hydroxytoluene (BHT), butylated hydroxyanisole (BHA), and tertiary butylhydroquinone (TBHQ), are used to retard the oxidation process. Investigations have demonstrated that synthetic antioxidants are toxic and carcinogenic for humans (Rehman et al. 2004). Therefore, there is a need to use natural antioxidants as alternatives to synthetic types. Studies showed that plants extracts are a rich source of bioactive compounds with strong antioxidant activity.

Loquat (*Eriobotrya japonica*) is an evergreen tree of Rosaceae family with short branches. The species is native to southeastern China and mainly grow on subtropical and in mild temperature. Currently, it is also cultivated in other areas, for instance, South Africa, South America, Australia, and California. Loquat flowers in late autumn or early winter and its fruit ripens in late winter or early spring (Ferreres et al. 2009). Loquat fruit grows in clusters and is circular with a smooth or downy form, and the skin color is yellow. Moreover, it has white, yellow, or orange pulp. Also, the fruit has three to five brown seeds. It tastes sour and sweet (meles) or sour which is dependent on the area where it grows (Xu et al. 2014). Loquat has good antioxidant property due to the presence of phenolic (benzoic acid and hydroxyl cinnamic derivatives) and tocopherol compounds (Xu and Chen 2011).

Recently, different methods are used for the extraction of plants bioactive compounds, such as supercritical fluid, microwaves, and ultrasound-assisted extraction. Although supercritical fluid CO_2_ extracts the nonpolar compounds, it is not able to isolate the polar compounds (Hamburger et al. 2004). So, the modifier solvents such as ethanol, acetone, and methanol are used to increase the extraction of polar compounds effectively (Lang and Wai 2001). Ultrasound waves during cavitation destroy plant cell wall and cause the extraction of more bioactive compounds. Sometimes the ultrasound waves cause damage to and reduce the yield of antioxidant compounds (Cao et al. 2009). Therefore, more research is required to determine which method is suitable for extraction of bioactive compounds of any plant.

The objective of this study was to evaluate the effects of different extraction methods (solvent, ultrasound-assisted, and supercritical fluid CO_2_ extraction) on the antioxidant activity of loquat fruit skin extract during oxidation of soybean oil by measuring both primary and secondary oxidation products and to compare its antioxidant activity with TBHQ.

## Materials and Methods

### Materials

Loquat fruits were collected from fields in Sari in the Mazandaran province (Iran). Refined, bleached, and deodorized soybean oil with no added antioxidants was supplied by Rana (Kordkouy, Iran) and was stored at −20°C until further analysis. All chemical materials and solvents which are used are analytical grade and are purchased from Merck (Darmstadt, Germany) or Sigma-Aldrich (St. Louis, USA).

### Sample preparation

Loquat fruit skins were separated and dried in the shade in natural conditions. Dried powders of sample (20 g) were mixed with 100 mL of ethanol. The mixture was stirred in a shaker (LABTRON Ls-100, Tabriz, Iran) at 160 rpm away from light at room temperature for 48 h. The extract was filtered and solvent evaporated using a rotary evaporator (Heidolph, Germany) at 50°C. The extract was stored at −20°C until testing time (Tachakittirungrod et al. 2007).

An Elma Transsonic ultrasonic bath model 690/H (Cottbus, Germany) was used for sonication and extraction of bioactive compounds from mixture of solvent and powdered skin. Dried powders of sample (20 g) were mixed with 100 mL of ethanol, then placed in ultrasonic bath, and then sonicated at 35 kHz for 30 min at 35°C. The extract was filtered and subsequently evaporated using a rotary evaporator. The concentrated extracts were stored in a freezer (Luengthanaphol et al. 2004).

A Suprex MPS/225 Multipurpose system (Roth Scientic, Basingstoke, Hampshire, U.K.) in the supercritical fluid CO_2_ extraction mode was used for the extraction of bioactive compounds. In this method, extraction of 20 g dried powder from the skin was accomplished with 100 mL ethanol at 35°C, 100 bar, for 30 min. The extract was filtered and concentrated using a rotary evaporator. The concentrated extract was stored under refrigeration until further analysis (Albu et al. 2004).

### Oil oxidation

The two concentrations of 400 and 1000 ppm for the oil samples were prepared by dissolving loquat fruit skin extracts in soybean oil. Oil samples were stored at 25°C in dark for 60 days. At the end of each 15 days (0, 15, 30, 45, and 60), about 20 g of the oil samples was filtered into a screw-cap vial and promptly stored in freezer at −20°C until further use (Rehman et al. 2004).

### Total phenolic content

The total phenolic content (TP) of extracts was determined using Folin–Ciocalteu reagent according to the procedure reported by McDonald et al. (2001). Briefly, 0.5 mL of extract was mixed with 2.5 mL of 10-fold-diluted Folin–Ciocalteu reagent and 2 mL of 7.5% sodium carbonate. Then, the mixture was shaken for 1 min and allowed to stand at room temperature for 15 min. Absorbance of the solution was measured at 765 nm using a spectrophotometer (Cintra 20; GBC, Dandenong, Australia). The concentration of phenolic compounds was estimated using a calibration curve traced with gallic acid (GA) in methanol at concentrations of 0.04–0.4 mg/mL as a polyphenol reference. The results are expressed as microgram of GA equivalent per milliliter extract (*μ*g GAE/mL). Each test was repeated three times, and the results were averaged.

### Total tocopherol content

The total tocopherol content (TT) was determined according to the colorimetric method described by Wong et al. (1988). A calibration curve of pure *α*-tocopherol in toluene was performed in a concentration range of 0–240 *μ*g/mL. Extract (0.2 g) was dissolved in 5 mL of toluene and then 3.5 mL of 2, 2′-bipyridine (0.07% w/v in 95% aqueous ethanol) and 0.5 mL of FeCl_3_-6H_2_O (0.2% w/v in 95% aqueous ethanol) were added into that mixture. The solution was made up to 10 ml with 95% aqueous ethanol. After standing for 1 min, the absorption at 520 nm was determined using a spectrophotometer. The results are expressed as microgram of *α*-tocopherol equivalents per milliliter extract (*μ*g *α*-tocopherol/mL).

### Peroxide value

The spectrophotometric method described by Shantha and Decker (1993) was used to determine peroxide value (PV). Briefly, 0.2 g of oil sample was dissolved in 9.8 mL chloroform–methanol (7:3 v/v). Fifty microliter of ammonium thiocyanate solution (30% w/v) was added and the sample mixed on a vortex mixer for 2–4 sec. Then, 50 *μ*L of iron (II) chloride solution ([0.4 g barium chloride dihydrate dissolved in 50 mL H_2_O] + [0.5 g FeSO_4_-7H_2_O dissolved in 50 mL H_2_O] + [2 mL 10 mol/L HCl, with the precipitate, barium sulfate, filtered off to produce a clear solution]) was added, and the sample mixed on a vortex mixer for 2–4 sec. After 5 min of incubation at room temperature, the absorbance of the sample was read at 500 nm against a blank that contained all the reagents except the sample, by using a spectrophotometer. Results are expressed in milliequivalents of oxygen per kilogram of oil.

### Thiobarbituric acid (TBA) value

This value was measured according to the method described by Senevirathne et al. (2006). Oil samples (1 g) were dissolved in 3.5 mL cyclohexane. Then, 4.5 mL of trichloroacetic acid (TCA 7.5%)–thiobarbituric acid (TBA 0.34%) mixture was added. Final mixture was shaken for 5 min and centrifuged for 15 min at 2780*g*. TCA-TBA phase was removed and heated in a boiling water bath for 10 min. Finally, absorbance was read at 532 nm by spectrophotometry. TBA concentration was estimated using a calibration curve traced based on 1,1,3,3-tetra-ethoxypropane. Results are expressed as equivalent *μ*mol of malondialdehyde per kg oil. Each test repeated three times and results were averaged.

### Conjugated dienes and trienes

Contents of conjugated dienes (CD) and conjugated trienes (CT) were calculated according to the method described by Fathi et al. (2013) which is based on the measurement of solution absorbance (5 mg of oil sample dissolved in 10 mL cyclohexane) at 234 and 270 nm for CD and CT, respectively.

### Free fatty acid content

The free fatty acid (FFA) content was determined using an alkali titration method according to Farhoosh et al. (2012). About 10 g of oil samples was dissolved in a 50-mL mixture of neutral ethanol–chloroform (50:50 v/v). Then, mixture was shaken by hand. Mixture was titrated against potassium hydroxide (0.1 N) using phenolphthalein solution (10 g/L) as an indicator. FFA value (%) was calculated according to the following equation: [FFA] = (*V* × *C* × 56.11)/m, where *V* is the volume of potassium hydroxide exhausted by samples (mL); *C* is the concentration of potassium hydroxide (mol/L); and *m* is the mass of soybean oil (g).

### Statistical analysis

Statistical analysis was performed using SPSS version 19.0 software (SPSS Inc., Chicago, IL, USA). All experiments were conducted in three levels of measurements and results were reported as mean ± standard deviation (SD). Data were subjected to analysis of variance (ANOVA). Duncan's test was used to determine significant differences between means of treatment (*P* < 0.05).

## Results and Discussion

### The total phenolic (TP) and tocopherol (TT) content

Many researches have shown that the antioxidant activities of phenolic and tocopherol compounds are probably due to their redox properties, which allow them to act as reducing agents, hydrogen donors, and singlet oxygen quenchers (Chang et al. 2001). The TP contents in extracts extracted by ultrasound-assisted, supercritical CO_2_, and solvent extraction methods were 394.67 ± 4.01, 425.02 ± 0.02, and 664.53 ± 2.59 *μ*g GAE per mL and the TT content was 388.58 ± 0.25, 228.31 ± 0.15, and 486.53 ± 0.67 *μ*g *α*-tocopherol per mL, respectively. Therefore, the solvent extraction method was more effective for extraction of tocopherol and phenolic compounds compared to other methods. Luengthanaphol et al. (2004) and Goli et al. (2005) reported that the solvent extraction was the most effective method in extraction of phenolic compounds to compare ultrasound-assisted and supercritical CO_2_ extraction methods. Already, a wide variation was observed on total phenolic content in fruits of loquat cultivars that ranged from 129 to 578 *μ*g GAE per g in Turkey (Polat et al. 2010), 240 to 572 *μ*g GAE per g in China (Xu and Chen 2011), and 125.7 to 2603.3 *μ*g GAE per g in Spain (Ferreres et al. 2009). According to Rop et al. (2011) and Milivojevic et al. (2012), the phenolic content and composition of fruits and vegetables depend on the genetic and environmental factors as well as postharvest processing conditions.

### Effect on PV

The PV is a measurement for the concentrations of peroxides and hydroperoxides produced in the first stage of oxidation of oils and fats (Zia-ur-Rehman 2006). The initial PV was the same as in oil samples (*P* < 0.05). The PV of control oil sample without any additives (SBO) reached to a maximum value of 31.15 meq O_2_ per kg after 65 days of storage (Table1). The PVs of TBHQ, SEA, SEB, SUA, SUB, SSB, and SSA were 9.42, 9.72, 10.06, 11.11, 11.82, 12.05, and 12.33 meq O_2_ per kg, respectively. It was generally observed that the extracts reduced PV compared to control oil significantly (*P* < 0.05). Results also showed that the PV increased linearly with increasing of storage days. Similar results have been reported by Rehman et al. (2004), Suja et al. (2005), and Goli et al. (2005). Trend of increase in PV of oils consisting solvent extract of skin was lower than other oils treated with extracts. There was no significant difference between the PV level of SEB at day 15 and SEA at days 30, 45 with TBHQ. Therefore, SEA showed a suitable antioxidant activity, but the best protection was observed in TBHQ. Sikwese and Duodu (2007) compared antioxidative effects of sorghum crude phenolic extract with TBHQ in sunflower oil in the presence of ferric ions. They showed that TBHQ made a higher inhibitory effect on primary oxidation of the oil than the extract and also Mohdaly et al. (2011) compared antioxidative effects of sesame cake extract with BHT, BHA, and TBHQ in soybean and sunflower oil. They showed that sesame cake extract exhibited stronger antioxidant activity in oils than BHT and BHA, while its antioxidant activity was less than that of TBHQ.

**Table 1:** Change in peroxide value (PV) of the oil samples during storage at 25 °C

	PV (meqO_2_/kg)							
Time (day)	SEA	SEB	SSA	SSB	SUA	SUB	TBHQ	SBO
0	0.22 ± 0.01a	0.22 ± 0.01a	0.22 ± 0.01a	0.22 ± 0.01a	0.22 ± 0.01a	0.22 ± 0.01a	0.22 ± 0.01a	0.22 ± 0.01a
15	2.12 ± 0.03c	1.45 ± 0.02a	2.35 ± 0.03e	2.14 ± 0.03c	2.22 ± 0.02d	1.85 ± 0.04b	1.42 ± 0.02a	5.85 ± 0.03f
30	4.63 ± 0.03a	5.37 ± 0.06b	6.54 ± 0.03d	6.88 ± 0.03e	5.42 ± 0.04b	5.68 ± 0.04c	4.57 ± 0.05a	12.77 ± 0.05f
45	6.75 ± 0.01a	7.24 ± 0.01b	8.52 ± 0.02e	8.84 ± 0.02f	7.32 ± 0.03c	7.77 ± 0.02d	6.73 ± 0.03a	20.64 ± 0.04g
60	9.72 ± 0.03b	10.06 ± 0.03c	12.33 ± 0.03g	12.05 ± 0.04f	11.11 ± 0.03d	11.82 ± 0.02e	9.45 ± 0.03a	31.15 ± 0.03h

Means ± SD within each row followed by different letters (a, b, c, etc.) are significantly different (*P* < 0.05). SEA, soybean oil with 400 ppm of solvent extract; SEB, soybean oil with 1000 ppm of solvent extract; SSA, soybean oil with 400 ppm of supercritical fluid CO_2_ extract; SSB, soybean oil with 1000 ppm of supercritical fluid CO_2_ extract; SUA, soybean oil with 400 ppm of ultrasound-assisted extract; SUB, soybean oil with 1000 ppm of ultrasound-assisted extract; TBHQ, soybean oil with 100 ppm of TBHQ; SBO, soybean oil with no antioxidant added.

### Effect on TBA value

Table2 shows change in TBA value of the oil samples during storage. TBA value gives a measure of lipid oxidation development, in terms of secondary oxidation products. This method is based on measuring pink complex formed at an absorbance of 532 nm after reaction of one molecule of malondialdehyde (MDA) with two molecules of TBA (Taghvaei et al. 2014). As it has been shown in Table2, TBA value of oil samples increased gradually during storage, similar studies were conducted by Goli et al. (2005), Zhang et al. (2010), and Kamkar et al. (2013). The TBA value of all soybean oils consisting loquat skin extracts had lower increasing trend, whereas TBA value for control oil samples reached from 0.05 to maximum of 0.4 *μ*mol per g MDA. Based on TBA value results, the oxidative stability of oil samples during 60 days of storage were as following order:

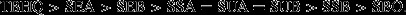


**Table 2:** Change in thiobarbituric acid value (TBA) of the oil samples during storage at 25°C

	TBA (*μ*mol/g)							
Time (day)	SEA	SEB	SSA	SSB	SUA	SUB	TBHQ	SBO
0	0.05 ± 0.02a	0.05 ± 0.02a	0.05 ± 0.02a	0.05 ± 0.02a	0.05 ± 0.02a	0.05 ± 0.02a	0.05 ± 0.02 a	0.05 ± 0.02a
15	0.105 ± 0.002ab	0.108 ± 0.002b	0.112 ± 0.001c	0.118 ± 0.003d	0.102 ± 0.001a	0.108 ± 0.002b	0.102 ± 0.001a	0.154 ± 0.002e
30	0.113 ± 0.002a	0.115 ± 0.003ab	0.122 ± 0.003c	0.128 ± 0.003d	0.119 ± 0.001b	0.129 ± 0.004d	0.113 ± 0.002a	0.225 ± 0.003e
45	0.132 ± 0.001b	0.135 ± 0.001b	0.145 ± 0.002cd	0.148 ± 0.004d	0.135 ± 0.001b	0.142 ± 0.003c	0.128 ± 0.002a	0.334 ± 0.002e
60	0.145 ± 0.004b	0.155 ± 0.004c	0.162 ± 0.002cd	0.165 ± 0.004d	0.158 ± 0.006cd	0.162 ± 0.002cd	0.135 ± 0.004a	0.402 ± 0.005e

Means ± SD within each row followed by different letters (a, b, c, etc.) are significantly different (*P* < 0.05). SEA, soybean oil with 400 ppm of solvent extract; SEB, soybean oil with 1000 ppm of solvent extract; SSA, soybean oil with 400 ppm of supercritical fluid CO_2_ extract; SSB, soybean oil with 1000 ppm of supercritical fluid CO_2_ extract; SUA, soybean oil with 400 ppm of ultrasound-assisted extract; SUB, soybean oil with 1000 ppm of ultrasound-assisted extract; TBHQ, soybean oil with 100 ppm of TBHQ; SBO, soybean oil with no antioxidant added.

After TBHQ, SEA showed the best antioxidant activity higher than that other oils treated with extracts. The TBA results of oil samples were concurred with previously published results such as the works by Monfared et al. (2011) and Kamkar et al. (2013).

### Effect on CD and CT values

Change in CD and CT values of the oil samples is shown in Tables3 and 4. Assessment of CD and CT values is suitable tests for determination of primary and secondary compounds of oxidation, respectively (Rafiee et al. 2012). The CD and CT values increased linearly during storage, similarly Bouaziz et al. (2008) and Mohdaly et al. (2011). The CD and CT values for the SBO at the end of the 60 days of storage were greater than that of the oils treated with extracts and TBHQ. The CD and CT values of soybean oils consisting solvent extract of loquat fruit skin was lower in comparison with ultrasonic and supercritical fluid CO_2_ extracts. The increase in the CD was considerably higher compared to the CT, which will be specifically due to the high content of linoleic acid in the soybean oil (Liu and White 1992). The SEA with no significant difference with TBHQ indicated a greater ability to reduce the production of conjugated compounds, compared to other oil samples during storage conditions. Our results for CD and CT values were in agreement with previously published results such as the works of Mohdaly et al. (2010, 2011).

**Table 3:** Change in conjugated dienes (CD) of the oil samples during storage at 25 °C

	CD (mmol/L)							
Time (day)	SEA	SEB	SSA	SSB	SUA	SUB	TBHQ	SBO
0	2.25 ± 0.02a	2.25 ± 0.02a	2.25 ± 0.02a	2.25 ± 0.02a	2.25 ± 0.02a	2.25 ± 0.02a	2.25 ± 0.02a	2.25 ± 0.02a
15	2.42 ± 0.01a	2.54 ± 0.04b	2.76 ± 0.04d	2.82 ± 0.04e	2.65 ± 0.03c	2.82 ± 0.02e	2.40 ± 0.03a	2.64 ± 0.02c
30	2.62 ± 0.03b	2.78 ± 0.03c	2.84 ± 0.01d	3.01 ± 0.04f	2.84 ± 0.02d	2.95 ± 0.03e	2.56 ± 0.02a	3.58 ± 0.01g
45	2.84 ± 0.03a	2.92 ± 0.04b	3.04 ± 0.03c	3.15 ± 0.03d	3.05 ± 0.04c	3.20 ± 0.02d	2.82 ± 0.02a	4.02 ± 0.03e
60	3.05 ± 0.04a	3.44 ± 0.04c	3.58 ± 0.03d	3.72 ± 0.03e	3.25 ± 0.03b	3.44 ± 0.03c	3.05 ± 0.03a	4.85 ± 0.02f

Means ± SD within each row followed by different letters (a, b, c, etc.) are significantly different (*P* < 0.05). SEA, soybean oil with 400 ppm of solvent extract; SEB, soybean oil with 1000 ppm of solvent extract; SSA, soybean oil with 400 ppm of supercritical fluid CO_2_ extract; SSB, soybean oil with 1000 ppm of supercritical fluid CO_2_ extract; SUA, soybean oil with 400 ppm of ultrasound-assisted extract; SUB, soybean oil with 1000 ppm of ultrasound-assisted extract; TBHQ, soybean oil with 100 ppm of TBHQ; SBO, soybean oil with no antioxidant added.

**Table 4:** Change in conjugated trienes (CT) of the oil samples during storage at 25 °C

	CT (mmol/L)							
Time (day)	SEA	SEB	SSA	SSB	SUA	SUB	TBHQ	SBO
0	0.45 ± 0.01a	0.45 ± 0.01a	0.45 ± 0.01a	0.45 ± 0.01a	0.45 ± 0.01a	0.45 ± 0.01a	0.45 ± 0.01a	0.45 ± 0.01a
15	0.55 ± 0.02ab	0.58 ± 0.02bc	0.62 ± 0.02cd	0.58 ± 0.01bc	0.55 ± 0.03ab	0.62 ± 0.03cd	0.52 ± 0.03a	0.64 ± 0.02d
30	0.58 ± 0.02a	0.68 ± 0.03cd	0.7 ± 0.02cd	0.72 ± 0.03d	0.65 ± 0.02bc	0.66 ± 0.04bc	0.62 ± 0.02ab	0.72 ± 0.04d
45	0.68 ± 0.04a	0.72 ± 0.04ab	0.75 ± 0.02bc	0.77 ± 0.03bc	0.75 ± 0.03bc	0.78 ± 0.03c	0.68 ± 0.01a	0.85 ± 0.03d
60	0.75 ± 0.03a	0.77 ± 0.02a	0.82 ± 0.02b	0.88 ± 0.03c	0.83 ± 0.03b	0.85 ± 0.02bc	0.74 ± 0.03a	1.02 ± 0.01d

Means ± SD within each row followed by different letters (a, b, c, etc.) are significantly different (*P* < 0.05). SEA, soybean oil with 400 ppm of solvent extract; SEB, soybean oil with 1000 ppm of solvent extract; SSA, soybean oil with 400 ppm of supercritical fluid CO_2_ extract; SSB, soybean oil with 1000 ppm of supercritical fluid CO_2_ extract; SUA, soybean oil with 400 ppm of ultrasound-assisted extract; SUB, soybean oil with 1000 ppm of ultrasound-assisted extract; TBHQ, soybean oil with 100 ppm of TBHQ; SBO, soybean oil with no antioxidant added.

### Effect on FFA content

Table5 shows the changes in FFA content of the oil samples during storage period. The FFA is used as an indicator for assessment of oil deterioration during oxidative conditions (Zhang et al. 2010). There was no significant difference between the initial FFA of the oil samples (*P* < 0.05). The FFA increased gradually for all of oil samples during storage which is similar to the results reported by Rehman et al. (2004) and Chotimarkorn and Silalai (2008). Adding extracts reduced the amount of FFA compared to control oil. This value for TBHQ, SEA, SEB, SUA, SSA, SSB, SUB, and SBO after 65 days of storage was 0.34%, 0.44%, 0.52%, 0.52%, 0.56%, 0.65%, 0.65%, and 1.22%, respectively. Therefore, the FFA in SEA was lower than those other oils containing extracts, but it could not make reduction compared to TBHQ. The FFA results concurred with Rehman et al. (2004) and Zia-ur-Rehman (2006) which examined the antioxidant effect of citrus peel extract and potato peels extract in soybean oil at 25°C.

**Table 5:** Change in free fatty acids content (FFA) of the oil samples during storage at 25°C

	FFA (%)							
Time (day)	SEA	SEB	SSA	SSB	SUA	SUB	TBHQ	SBO
0	0.06 ± 0.01a	0.06 ± 0.01a	0.06 ± 0.01a	0.06 ± 0.01a	0.06 ± 0.01a	0.06 ± 0.01a	0.06 ± 0.01a	0.06 ± 0.01a
15	0.15 ± 0.01c	0.15 ± 0.02c	0.22 ± 0.02d	0.11 ± 0.02b	0.22 ± 0.02d	0.11 ± 0.02b	0.07 ± 0.01a	0.28 ± 0.03e
30	0.22 ± 0.02b	0.34 ± 0.02c	0.34 ± 0.03c	0.44 ± 0.02e	0.38 ± 0.03cd	0.44 ± 0.02e	0.11 ± 0.03a	0.42 ± 0.02de
45	0.34 ± 0.01b	0.44 ± 0.01c	0.44 ± 0.04c	0.52 ± 0.02d	0.44 ± 0.01c	0.52 ± 0.03d	0.22 ± 0.02a	0.75 ± 0.01e
60	0.44 ± 0.04b	0.52 ± 0.01c	0.56 ± 0.03c	0.65 ± 0.02d	0.52 ± 0.02c	0.65 ± 0.01d	0.34 ± 0.01a	1.22 ± 0.02e

Means ± SD within each row followed by different letters (a, b, c, etc.) are significantly different (*P* < 0.05). SEA, soybean oil with 400 ppm of solvent extract; SEB, soybean oil with 1000 ppm of solvent extract; SSA, soybean oil with 400 ppm of supercritical fluid CO_2_ extract; SSB, soybean oil with 1000 ppm of supercritical fluid CO_2_ extract; SUA, soybean oil with 400 ppm of ultrasound-assisted extract; SUB, soybean oil with 1000 ppm of ultrasound-assisted extract; TBHQ, soybean oil with 100 ppm of TBHQ; SBO, soybean oil with no antioxidant added.

## Conclusion

In this study, it can be concluded that the solvent extraction method was more effective on antioxidant activity of loquat fruit skin extract compared to ultrasound-assisted and supercritical fluid CO_2_ extraction methods in stability of soybean oil during 60-day storage at 25°C. Therefore, it is suggested that the best method for the extraction of antioxidant containing tocopherol and phenolic compounds is by solvent extraction method. According to PV, FFA, TBA, CD, and CT values, the SEA indicated an adequate antioxidant activity, but the best protection was observed in soybean oil consisting TBHQ. These results suggest that the loquat fruit skin extract can be used as a natural antioxidant to improve the quality, stability, and safety of foods such as edible oils.

## Conflict of Interest

None declared
