# Antioxidative effect of loquat (*Eriobotrya japonica* Lindl.) fruit skin extract in soybean oil

**DOI:** 10.1002/fsn3.319

**Published:** 2015-11-15

In Delfanian et al. (2015), the following errors were published in the affiliation and correspondence details of the first author, Mojtaba Delfanian.

**Author byline and affiliation**

Mojtaba Delfanian^1^, Reza Esmaeilzadeh Kenari^1^ & Mohammad Ali Sahari^2^

^1^Department of Food Science and Technology, Sari Agriculture and Natural Resources University, Sari, Mazandaran, Iran

^2^Department of Food Science and Technology, Faculty of Agriculture, Tarbiat Modares University, Tehran, Iran

The affiliations were incorrect and should have read:

**Author byline and affiliation**

Mojtaba Delfanian^1^, Reza Esmaeilzadeh Kenari^2^ & Mohammad Ali Sahari^3^

^1^Graduated from Faculty of Food Science and Technology, Gorgan University of Agricultural Sciences and Natural Resources, Gorgan, Iran

^2^Department of Food Science and Technology, Sari Agriculture and Natural Resources University, Sari, Mazandaran, Iran

^3^Department of Food Science and Technology, Faculty of Agriculture, Tarbiat Modares University, Tehran, Iran

**Correspondence section**

Mojtaba Delfanian, Department of Food Science and Technology, Sari Agriculture and Natural Resources University, P.O. Box 578, Sari, Mazandaran, Iran. Tel: +989355302182; Fax: +981132203357; E‐mail: makan.delfan@yahoo.com

The text was incorrect and should have read:

**Correspondence section**

Mojtaba Delfanian, Graduated from Faculty of Food Science and Technology, Gorgan University of Agricultural Sciences and Natural Resources, Gorgan, Iran. Tel: +989355302182; Fax: +981133115155; E‐mail: makan.delfan@yahoo.com

We apologize for these errors.
